# Rare Earth Extraction from NdFeB Magnet Using a Closed-Loop Acid Process

**DOI:** 10.1038/s41598-017-08629-z

**Published:** 2017-08-14

**Authors:** Jiro Kitagawa, Ryohei Uemura

**Affiliations:** Department of Electrical Engineering, Faculty of Engineering, Fukuoka Institute of Technology, 3-30-1 Wajiro-higashi, Higashi-ku, Fukuoka 811-0295 Japan

## Abstract

There is considerable interest in extraction of rare earth elements from NdFeB magnets to enable recycling of these elements. In practical extraction methods using wet processes, the acid waste solution discharge is a problem that must be resolved to reduce the environmental impact of the process. Here, we present an encouraging demonstration of rare earth element extraction from a NdFeB magnet using a closed-loop hydrochloric acid (HCl)-based process. The extraction method is based on corrosion of the magnet in a pretreatment stage and a subsequent ionic liquid technique for Fe extraction from the HCl solution. The rare earth elements are then precipitated using oxalic acid. Triple extraction has been conducted and the recovery ratio of the rare earth elements from the solution is approximately 50% for each extraction process, as compared to almost 100% recovery when using a one-shot extraction process without the ionic liquid but with sufficient oxalic acid. Despite its reduced extraction efficiency, the proposed method with its small number of procedures at almost room temperature is still highly advantageous in terms of both cost and environmental friendliness. This study represents an initial step towards realization of a closed-loop acid process for recycling of rare earth elements.

## Introduction

Demand for NdFeB magnets is growing rapidly for applications including electric motor vehicles and wind turbines^1^, which offer sustainability and/or low-carbon emissions. Recently, recycling of the rare earth elements that can be extracted from used magnets has become an important research area because of issues with declining natural resources^2–5^. Countries with limited resources are urgently using recycling methods to provide stocks of these materials. Following the development of ore dressing technologies, a wet processing method^6^ has already been applied to recycling of the sludge from in-plant scrap. After acid leaching of the scrap using HCl, HNO_3_, and H_2_SO_4_ and filtration of the insoluble material, which mainly contains Fe, the resulting acid solution is reacted with either oxalic or carbonic acid to form a precipitate that contains the rare earth elements. The calcined precipitate forms rare earth oxides, which can then be returned to the initial manufacturing process for NdFeB magnets.

Roasting of the NdFeB magnet^7–9^ as a pretreatment process improves the selectivity between the rare earth elements and Fe, but the rare earth element recovery ratio is usually rather low because of acid leaching (particularly with HCl) at room temperature. Almost 100% recovery is achieved^7, 8^ when the HCl solution is heated to 80–180 °C. In the acid leaching method for sludge treatment^10^, it is also necessary to heat the acid solution to 80 °C. We have recently proposed a pretreatment involving corrosion of the magnet^11^ before dissolution in HCl and the oxalic acid precipitation process. The Nd recovery ratio in this process reaches 97%, even when a room temperature process is used, which represents the major advance of this method.

From the perspectives of sustainability and ecology, the main issue with this wet process is discharge of the waste acid solution. The waste acid’s recyclability is critically dependent on efficient extraction of the constituent elements of the magnet from the used acid. One promising method for Fe extraction involves reaction of the solution with an ionic liquid^7, 12–15^, which often has high selectivity between rare earth elements and Fe. Trihexyl(tetradecyl)phosphonium chloride (Cyphos® IL101) is a well-characterized ionic liquid that can extract Fe^3+^ ions from HCl solutions with zero extraction of the trivalent rare earth ions^7, 13^. A possible closed-loop acid process for use on a roasted NdFeB magnet has also been proposed, and the elemental technologies have been investigated thoroughly^7^. However, no actual demonstration involving reuse of the waste acid solution has been performed to date.

**Figure 1:**
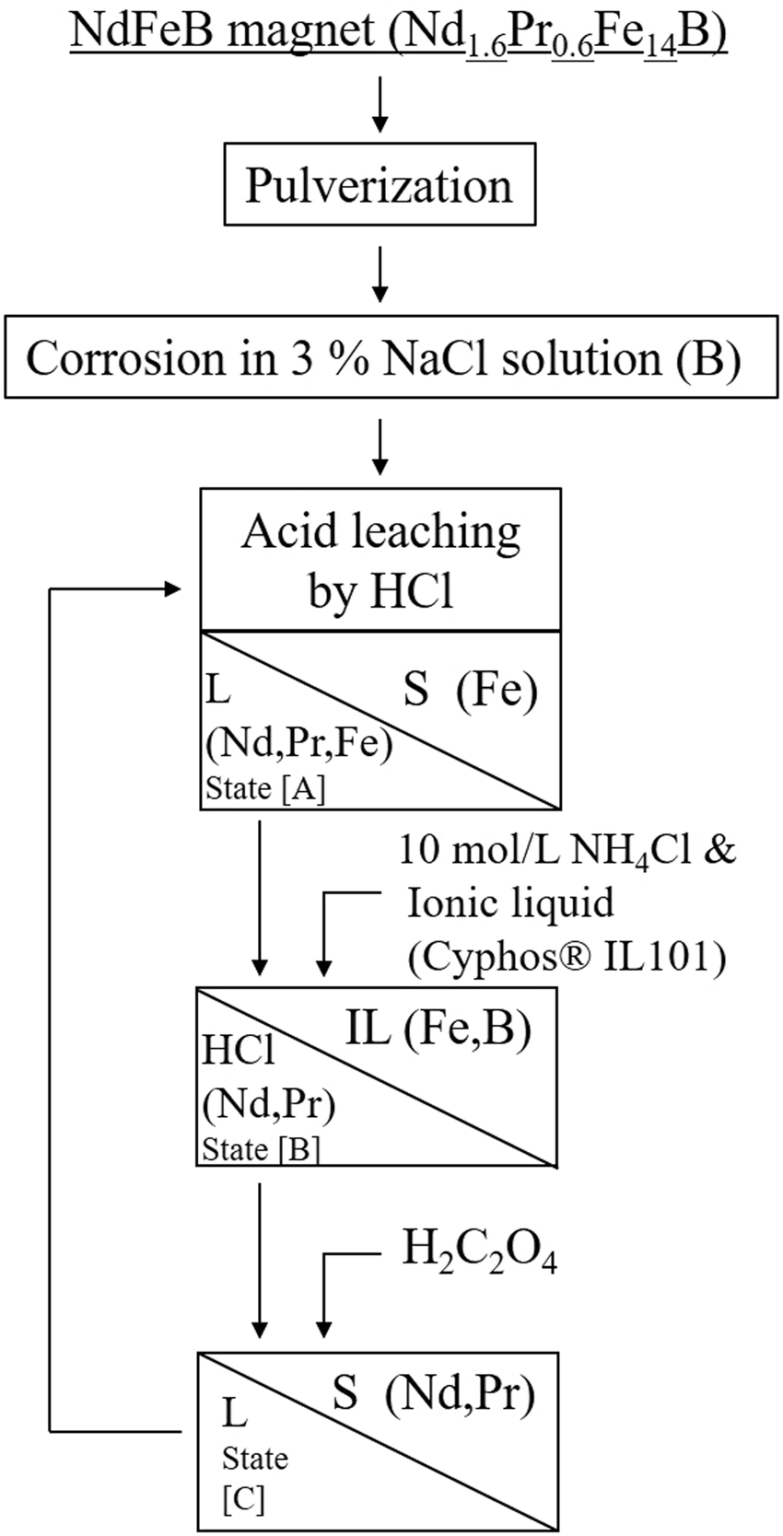
Procedures for rare earth element recovery from NdFeB magnet. S, L and IL denote the solid, the liquid and the ionic liquid, respectively. In each procedure, the elements that are expected to be present in the solid or solution are indicated.

If Cyphos® IL101 works well when used in our method, we may then demonstrate a closed-loop acid process that operates near room temperature, which would be highly advantageous. Figure 1 shows the work flow of the improved rare earth element extraction process without discharge of the waste HCl solution. Fe is partially dissolved in the HCl solution as Fe^3+^ after acid leaching of the corroded magnet. The ionic liquid efficiently extracts these Fe^3+^ ions using NH_4_Cl as a salting-out agent. After separation of the rare earth ions through an oxalic acid precipitation process, the HCl solution can then be reused for a subsequent extraction. In this report, we demonstrate that reuse of the HCl solution in a near-room temperature process is feasible when using our method.

## Results

**Figure 2:**
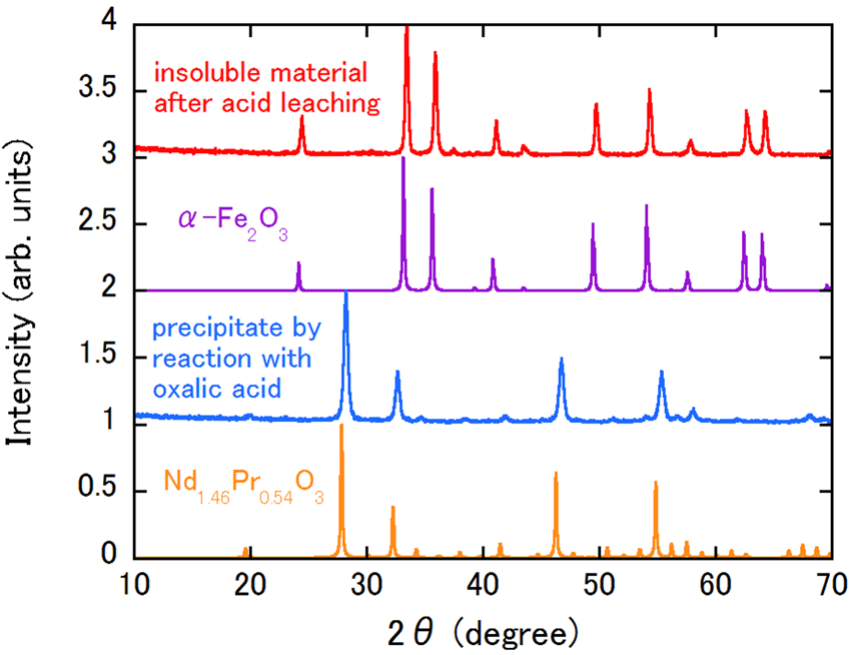
XRD patterns of calcined insoluble materials after acid leaching and oxalic acid precipitation processes. Simulated XRD patterns of α-Fe_2_O_3_ and Mn_2_O_3_-type Nd_1.46_Pr_0.54_O_3_ are also shown. The origin of each pattern has been shifted by an integer value for clarity.

The composition of the initial magnet, which has a mass of approximately 0.5 g, is Nd_1.6_Pr_0.6_Fe_14_B. The constituent element distribution in our method is unknown without the use of an ionic liquid. Therefore, a one-shot extraction procedure using 0.2 mol/L of HCl and 0.26 g of oxalic acid was performed. Table 1 shows the distributions of each of the ions in the NaCl solution after removal of the corroded sample and the corresponding distributions in the HCl solution after removal of the precipitates produced by reaction with oxalic acid. In the NaCl solution, only B is detected. Use of a sufficient amount of oxalic acid can separate the rare earth elements. A large quantity of Fe remains in the HCl solution. The ion concentrations that are expected for a completely dissolved 0.5 g magnet are 1041 mg/L for Nd, 381 mg/L for Pr, 3527 mg/L for Fe and 49 mg/L for B. The sum of the B concentrations in the two states listed in Table 1 is close to 49 mg/L. Approximately 30% of the B content can then be separated using the NaCl solution, while the remaining B content stays in the HCl solution. Approximately 60% of the Fe ions are contained in the insoluble material that is obtained after acid leaching. The recovery ratios of Nd and Pr are 99% and 97%, respectively. We have also checked that the calcined insoluble material that is obtained after acid leaching and the oxalic acid precipitate contain only Fe and rare earth elements, respectively (see Fig. 2). The X-ray diffraction (XRD) patterns of both samples show good agreement with those of α-Fe_2_O_3_ and Mn_2_O_3_-type Nd_1.46_ Pr_0.54_O_3_, respectively.

**Table 1:** Distributions of Nd, Pr, Fe and B in the one-shot recovery process. ND means not detected.

Solution	Nd (mg/L)	Pr (mg/L)	Fe (mg/L)	B (mg/L)
NaCl after removal of corroded magnet	ND	ND	ND	12.3
HCl after removal of oxalic acid precipitates	10.6	13.0	1480	28.6

A closed-loop acid process requires adjustment of both the salting-out agent’s NH_4_Cl concentration and the amount of oxalic acid used (see also Fig. 1). The salting-out agent is required for complete extraction of Fe from the HCl solution^7^. Adjustment of the amount of oxalic acid is important to prevent immediate precipitation of the rare earth elements by excess oxalic acid from the previous cycle. From preliminary experiments for the closed-loop process using the HCl solution (see Supplementary Information), we obtained the experimental requirements for 10 mol/L of NH_4_Cl and 0.1675 g of oxalic acid for recycling of a 0.5 g magnet.

**Figure 3:**
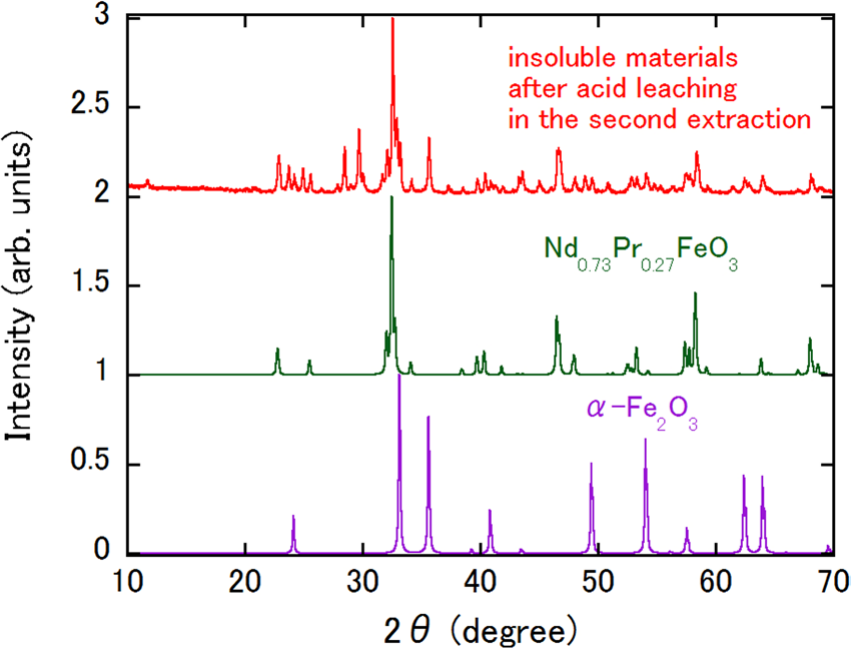
XRD patterns of calcined insoluble materials after acid leaching in the second extraction procedure. Simulated Nd_0.73_Pr_0.27_FeO_3_ and α-Fe_2_O_3_ patterns are also shown. The origin of each pattern has been shifted by an integer value for clarity.

Table 2 shows the results of a demonstration of the process of triple rare earth element extraction from a 0.5 g magnet using a closed-loop HCl system. The HCl concentration of 0.5 mol/L was used to expedite the experiment. Hereafter, the HCl solutions at the stages after removal of the insoluble material produced by acid leaching, after Fe extraction using the ionic liquid, and after removal of the rare earth elements by oxalic acid precipitation are called states [A], [B] and [C], respectively (see also Fig. 1). The concentrations of the Nd and Pr ions are measured in each of states [A], [B] and [C]. In each cycle, 10–15% of the available Nd and Pr ions are extracted together with the Fe ions by the ionic liquid, as demonstrated in the preliminary experiment (see Table S1). Contrary to our expectations, 30–35% of the Nd and Pr ions remained in solution after oxalic acid precipitation. However, these ions do not appear to contribute to the rare earth element concentrations of state [A] in the next cycle; the Nd and Pr ion concentrations in state [A] are the same as those measured during the previous cycle. The recovery ratio for each element, which is calculated by eliminating the amount of the relevant element that was extracted using the ionic liquid, is approximately 50% for all cycles. Figure 3 shows the XRD pattern of the calcined insoluble material after acid leaching during the second cycle. Simulated patterns for Nd_0.73_Pr_0.27_FeO_3_ and α-Fe_2_O_3_ are also shown. Elemental Nd and Pr are both partially recovered along with Fe. The fact that the XRD pattern in Fig. 3 contains Nd_0.73_Pr_0.27_FeO_3_ supports the idea that the Nd and Pr elements, which are present in the same concentrations as the ions in state [C], would then enter the insoluble material in state [A] in the next cycle.

**Table 2:** Distributions of Nd and Pr and recovery ratios for each element in a triple rare earth extraction process. The solution notations are as defined in Fig. 1.

Cycle	Solution state	Nd (mg/L)	Pr (mg/L)	Nd recovery ratio (%)	Pr recovery ratio (%)
1st	[A]	971	370	50	47
	[B]	818	311		
	[C]	328	136		
2nd	[A]	980	406	49	50
	[B]	836	357		
	[C]	352	156		
3rd	[A]	919	410	61	56
	[B]	842	360		
	[C]	280	130

**Figure 4:**
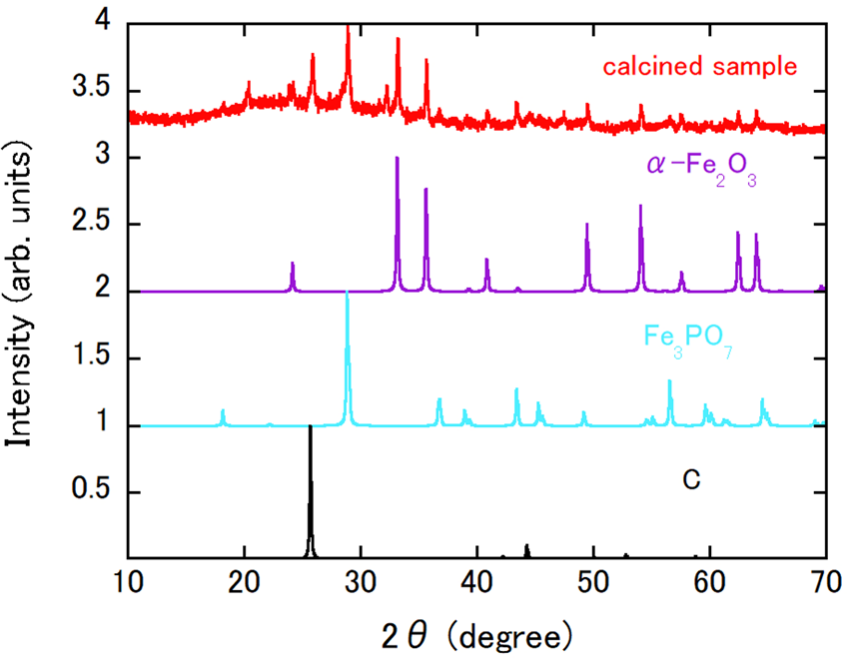
XRD pattern of calcined sample recovered from used Cyphos® IL101. Simulated α-Fe_2_O_3_, Fe_3_PO_7_ and C patterns are also shown. The origin of each pattern has been shifted by an integer value for clarity.

The ionic liquid used in our method, Cyphos® IL101, is comparatively expensive and regeneration of this ionic liquid by stripping of the Fe^3+^ ions is necessary to reduce the process cost. The elemental stripping technologies required have been reported previously^7, 14, 16^. For Cyphos® IL101, ammonia is a good candidate solution^16^ that approaches 100% stripping of Fe^3+^. We have also checked on the regeneration of used Cyphos® IL101 when roughly following a previously reported recipe^16^. Figure 4 shows the XRD pattern of the calcined sample that was recovered from used Cyphos® IL101. The figure also shows the simulated patterns for α-Fe_2_O_3_, Fe_3_PO_7_ and C. The experimental XRD pattern matches the superpositioned simulated patterns closely. The sources of P and C atom contamination would be the Cyphos® IL101 solution containing P and the cellulose filter, respectively. Despite the contamination that occurs as a result of our inadequate filtration technique, Fe can still be stripped. Additionally, the yellow-coloured ionic liquid became transparent once more after Fe^3+^ stripping, which means that the ionic liquid has been regenerated.

## Discussion

The demonstration of the closed-loop process using the HCl solution indicates that the precipitation process using oxalic acid alone is not sufficient, although the amount of oxalic acid used is higher than the ideal amount that was calculated using the chemical formula for precipitation (see Supplementary Information). In terms of actually increasing the rare earth element recovery ratios, if the amount of oxalic acid is increased, then it will cause a reduced recovery ratio in the second cycle, as can be deduced from Table S1. Therefore, a trade-off position between the number of rare earth element extractions and the recovery ratios of the rare earth elements may exist for the precipitation condition presented here.

The main cause of the reduced recovery ratio is insufficient ionization of the oxalic acid. The degree of ionization of oxalic acid is strongly dependent on the pH of the solution. The ionization concentration generally increases with increasing solution pH, and full ionization of the oxalic acid can be realized with an ideal mass of 0.1335 g (see Supplementary Information). Another issue that must be considered is partial rare earth extraction using the ionic liquid. If the oxalic acid precipitation process is performed before the Fe^3+^ extraction process using the ionic liquid, only the rare earth elements would be separated because of the high selectivity between the rare earth elements and Fe during oxalic acid precipitation. This issue can thus be resolved by reversing the order of the two processes in the sequence.

As shown in Fig. 3, unassigned peaks for materials other than α-Fe_2_O_3_ and Nd_0.73_Pr_0.27_FeO_3_ are also present in the XRD spectrum. In our study, complete separation of the ionic liquid from the HCl solution is difficult and this results in contamination of the calcined sample. Further improvements in the separation technique are therefore required to obtain a pure calcined sample.

Table 3 shows a comparison between our method and other reported methods^7–10^ with a specific focus on the one-shot recovery processes based on the acid leaching method that were mentioned in the Introduction. The number of steps in the recovery processes and the rare earth recovery ratios for each of the methods are similar. The roasting process used in the methods of refs 7, 8 and 9 would bring a cost disadvantage. While the method of ref. 10 does not use a roasting stage, the use of HNO_3_ is disadvantageous because waste discharges that contain nitrate salts are strictly controlled by law for environmental reasons. The acid leaching processes used in our method and in ref. 9 are performed at room temperature, which means that these are comparatively safe processes. Because the presence of B is harmful, its separation is highly desirable. Our method has achieved 30% B separation, whereas the other methods did not report clear B separation. If a closed-loop acid process with a high recovery ratio of rare earths is realized, our method is promising because each step apart from the Fe extraction process using the ionic liquid is performed at room temperature. This condition and the rather simple procedures required combine to provide a safe and low-cost recovery process. In addition, the B separation feature of our method is environmentally friendly.

**Table 3:** Comparison between proposed method and other methods based on acid leaching in one-shot recovery process. RT means room temperature.

Items	Proposed method (Ref. 11)	Ref. 7	Ref. 8	Ref. 9	Ref. 10
Pretreatment	Corrosion at RT	Roasting at 950 °C	Roasting at 500–1000 °C	Roasting at 750 °C	Sludge
Acid	HCl	HCl	HCl	H_2_SO_4_	HNO_3_
Acid leaching temperature	RT	80 °C	180 °C	RT	80 °C
B separation	30%	Not separated	Not separated	Not separated	Not separated
Recovery ratio of rare earth elements	97%	100%	100%	100%	94%

Rare earth element extraction methods based on acid leaching are entering the practical usage stage. For both sustainability and environmental reasons, the recyclability of the waste acid solution is one of the central issues in rare earth recycling, and this issue has not been investigated in depth to date. In this work, we have experimentally determined the recovery ratios of the rare earth elements in our method using a closed-loop acid process. This ratio is approximately 50%, as compared to the near-full recovery obtained in one-shot extraction methods. While this recovery ratio is rather low at the present stage, our encouraging results should lead to rapid and widespread development of studies of recycling using a closed-loop acid process.

## Methods

### Detailed process flow

A demagnetized and pulverized commercial NdFeB magnet (Niroku Seisakusyo) with mass of approximately 0.5 g was immersed in a 3% NaCl solution (300 mL) for 1 week. An air pump provided a constant air flow to the solution to accelerate the corrosion process. The corroded sample was then dissolved at room temperature in HCl solutions (100 mL) with concentrations of 0.2 mol/L or 0.5 mol/L for 1–2 h. After removal of any insoluble material, the solution was then reacted with the ionic liquid Cyphos® IL101 (HCl:ionic liquid = 4:1 in volume ratio), which was purchased from Sigma-Aldrich. A salting-out agent of 10 mol/L of NH_4_Cl was then added to the solution. The mixture was subjected to magnetic stirring at 750 rpm and 60 °C for 10 min. The mixture was then centrifuged at 2500 rpm for 10 min and split into each of its components. The HCl solution was then reacted with the oxalic acid.

### Stripping of Fe^3+^ from the ionic liquid

The yellow-coloured ionic liquid that contained Fe^3+^, which was obtained in the actual process flow, was reacted with an ammonia solution (approximately 3 wt.%) with a volume ratio of Cyphos® IL101:ammonia solution = 1:10. The resulting precipitate at the interface between the liquids was collected using a cellulose filter and was calcined with the filter at 800 °C for 5 h in air.

### Sample characterization

The composition of the demagnetized magnet was checked using an energy dispersive X-ray spectrometer that was equipped in a field emission scanning electron microscope (JEOL, JSM-7100F). Several samples, which had first been calcined at 800 °C for 5 h in air, were evaluated using a powder X-ray diffractometer (Shimadzu, XRD-7000L) with Cu-Kα radiation. We used an inductively coupled plasma atomic emission spectrometer (Shimadzu, ICPE-9000) to analyse the concentrations of Nd, Pr, Fe and B that were dissolved in the HCl or NaCl solutions. These concentrations were determined based on the working curves of standard Nd, Pr, Fe and B liquids.

## Electronic supplementary material

Supplementary Information

## References

[1] Goonan, T. G. Rare earth elements – end use and recyclability. *US Geological Survey Scientific Investigations Report 2011–5094*, 1–15 (U.S. Geological Survey, 2011).

[2] Tanaka M, Oki T, Koyama K, Narita H, Oishi T (2013). Recycling of rare earths from scrap. Handb. Phys. Chem. Rare Earths.

[3] Binnemans K (2013). Recycling of rare earths: a critical review. J. Clean. Prod..

[4] Darcy JW, Bandara D, Mishra B, Emmert MH (2013). Challenges in recycling end-of-life rare earth magnets. JOM.

[5] Rademaker JH, Kleijn R, Yang Y (2013). Recycling as a strategy against rare earth element criticality: a systemic evaluation of the potential yield of NdFeB magnet recycling. Environ. Sci. Technol..

[6] Xie F, Zhang TA, Dreisinger D, Doyle F (2014). A critical review on solvent extraction of rare earths from aqueous solutions. Miner. Eng..

[7] Hoogerstraete, T. V., Blanpain, B., Gerven, T. V. & Binnemans, K. From NdFeB magnets towards the rare-earth oxides: a recycling process consuming only oxalic acid. *RSC Adv.* **4**, 64099–64111 (2014).

[8] Japan Oil, Gas and Metals National Co., National Institute of Advanced Industrial Science and Technology & Tohoku Univ. Method of rare-earth extraction by acid leaching. *Japanese Unexamined Patent Application Publication* No. 2011-184735 (2011).

[9] Önal MAR, Borra CR, Guo M, Blanpain B, Van Gerven TV (2015). Recycling of NdFeB magnets using sulfation, selective roasting, and water leaching. J. Sustain. Metall..

[10] Rabatho JP, Tongamp W, Takasaki Y, Haga K, Shibayama A (2013). Recovery of Nd and Dy from rare earth magnetic waste sludge by hydrometallurgical process. J. Mater. Cycles Waste Manag..

[11] Kataoka Y, Ono T, Tsubota M, Kitagawa J (2015). Improved room-temperature-selectivity between Nd and Fe in Nd recovery from Nd-Fe-B magnet. AIP Advances.

[12] Billard I (2013). Ionic liquids: new hopes for efficient lanthanide/actinide extraction and separation?. Handb. Phys. Chem. Rare Earths.

[13] Vander Hoogerstraete TV, Wellens S, Verachtert K, Binnemans K (2013). Removal of transition metals from rare earths by solvent extraction with an undiluted phosphonium ionic liquid: separations relevant to rare-earth magnet recycling. Green Chem..

[14] Dupont D, Binnemans K (2015). Recycling of rare earths from NdFeB magnets using a combined leaching/extraction system based on the acidity and thermomorphism of the ionic liquid (Hbet)(Tf2N). Green Chem..

[15] Parmentier D, Vander Hoogerstraete TV, Metz SJ, Binnemans K, Kroon MC (2015). Selective extraction of metals from chloride solutions with the tetraoctylphosphonium oleate ionic liquid. Ind. Eng. Chem. Res..

[16] Wellens S (2014). Dissolution of metal oxides in an acid-saturated ionic liquid solution and investigation of the back-extraction behaviour to the aqueous phase. Hydrometallurgy.

