# Supplementary material for: Rare Earth Extraction from NdFeB Magnet Using a Closed-Loop Acid Process
Source: Sci Rep. 2017 Aug 14;7:8039. doi: 10.1038/s41598-017-08629-z (PMC5556000; doi:10.1038/s41598-017-08629-z)
Supplement: Supplementary file 1 — Supplementary Information [file 41598_2017_8629_MOESM1_ESM.pdf]

Supplementary Information

Rare Earth Extraction from NdFeB Magnet Using a Closed-Loop Acid Process

Jiro Kitagawa & Ryohei Uemura

Adjustment of concentration of salting-out agent NH_4Cl

Following the work in a previous paper[7] that reported that the salting-out agent NH_4Cl plays an essential role in full Fe extraction using an ionic liquid, the dependence of the extraction efficiency on the NH_4Cl concentration was determined. Without NH_4Cl, the Fe extraction efficiency was only 40%, but this efficiency increased to 75% when using 5 mol/L of NH_4Cl and to 95% when using 10 mol/L of NH_4Cl. In these experiments, we also found that B was fully extracted by the ionic liquid.

Preliminary experiment for closed-loop process using HCl solution

The preliminary experiment for the closed-loop process using the HCl solution was performed using 0.5 mol/L of HCl to reduce the time required for the experiment. The oxalic acid mass was maintained at 0.26 g. Hereafter, the HCl solutions obtained after removal of the insoluble material from the acid leaching, after Fe extraction using the ionic liquid, and after removal of the rare earth elements by oxalic acid precipitation are

denoted by states [A], [B] and [C], respectively (see also Fig. 1). Table S1 shows the Nd, Pr and Fe concentrations for each of these states during each cycle. In the first cycle, approximately 24% of the rare earth elements are extracted along with Fe using the ionic liquid. The oxalic acid precipitates Nd and Pr only. In state [A] during the second cycle, the Nd and Pr concentrations were approximately one-quarter of those during the first cycle, which indicates immediate precipitation due to the excess oxalic acid present in the previous cycle.

Table S1. Distributions of Nd, Pr and Fe in the preliminary experiment for the closed-loop process using the HCl solution. The solution notations are as defined in Fig. 1. ND means not detected.

Cycle	Solution state	Nd (mg/L)	Pr (mg/L)	Fe (mg/L)
**1st**	**[A]**	**929**	**346**	**2320**
	**[B]**	**707**	**264**	**105**
	**[C]**	**16.9**	**17.4**	**109**
**2nd**	**[A]**	**218**	**94**	**2770**
	**[B]**	**231**	**99**	**132**
	**[C]**	**ND**	**ND**	**134**

Adjustment of the mass of oxalic acid

The results of the preliminary experiment indicated that the mass of the oxalic acid should be adjusted. The mass of oxalic acid required for a 0.5 g magnet to reproduce the initial concentrations of Nd and Pr shown in state [A] in the second cycle has been researched, with results as shown in Fig. S1. The vertical axis shows the Nd (Pr) ion concentration difference in state [A] between the first and second cycles, which is denoted by Δc. A positive value for Δc indicates poor precipitation efficiency, while a negative value indicates excess oxalic acid. For each element, Δc decreases linearly with increasing oxalic acid mass. An oxalic acid mass of 0.1675 g can reproduce the initial Nd (Pr) ion concentration of state [A] in the second cycle.

The chemical equation for the oxalic acid precipitation process is

$$1.46Nd^{3+} + 0.54Pr^{3+} + 3H_2C_2O_4 \rightarrow (Nd_{0.73}Pr_{0.27})_2(C_2O_4)_3 + 3H_2\uparrow.$$

If the starting magnet mass is 0.5 g, the ideal quantity of oxalic acid is 0.1335 g. However, as shown in Fig. S1, Nd and Pr would not be fully precipitated by 0.1335 g of oxalic acid. To achieve sufficient precipitation, the quantity of oxalic acid that must be consumed must be 1.3 times greater.

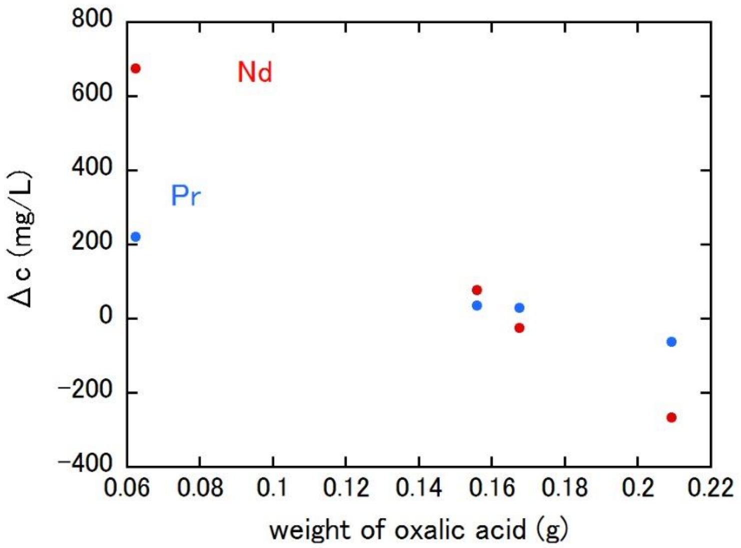

Figure S1. Plot of Δc vs. mass of oxalic acid. Δc represents the difference in Nd (Pr) ion concentration after acid leaching of a corroded magnet between the first and second cycles.
